# Supplementary material for: Transcriptomic Insights into Mechanisms of Early Seed Maturation in the Garden Pea (Pisum sativum L.)
Source: Cells. 2020 Mar 23;9(3):779. doi: 10.3390/cells9030779 (PMC7140803; doi:10.3390/cells9030779)

# Zhongwan−6(10)

## Global Summary

%DE = 0.86

# genes with fdr < 0.2 = 542 ( 204 + / 338 −)

# genes with fdr < 0.1 = 497 ( 180 + / 317 −)

# genes with fdr < 0.05 = 447 ( 154 + / 293 −)

# genes with fdr < 0.01 = 375 ( 106 + / 269 −)

# genes in genesets = 0

<FC> = 0

<t−score> = −701.56

<p−value> = 0

<fdr> = 0.15

## Global Genelist

| Rank | ID | log(FC) | p−value | fdr | Metagene | Description |
|---|---|---|---|---|---|---|
| 1 | comp22899_c | −0.19 | 3e−15 | 3e−12 | 1 x 5 | |
| 2 | TRINITY_DN4 | −0.15 | 4e−14 | 1e−11 | 5 x 2 | |
| 3 | TRINITY_DN6 | −0.11 | 3e−13 | 1e−11 | 2 x 3 | |
| 4 | TRINITY_DN6 | −0.13 | 3e−13 | 3e−11 | 1 x 4 | |
| 5 | TRINITY_DN6 | −0.1 | 2e−12 | 3e−11 | 6 x 20 | |
| 6 | TRINITY_DN4 | −0.75 | 2e−12 | 3e−11 | 1 x 1 | |
| 7 | comp16243_c | −0.01 | 2e−12 | 3e−11 | 6 x 8 | |
| 8 | TRINITY_DN6 | −0.05 | 3e−12 | 3e−11 | 6 x 6 | |
| 9 | TRINITY_DN1 | −0.21 | 3e−12 | 3e−11 | 1 x 3 | |
| 10 | TRINITY_DN3 | −0.03 | 3e−12 | 3e−11 | 8 x 15 | |
| 11 | TRINITY_DN6 | −0.12 | 3e−12 | 3e−11 | 16 x 9 | |
| 12 | TRINITY_DN4 | −0.08 | 4e−12 | 3e−11 | 7 x 20 | |
| 13 | TRINITY_DN1 | −0.03 | 4e−12 | 3e−11 | 5 x 15 | |
| 14 | TRINITY_DN5 | −0.03 | 4e−12 | 3e−11 | 13 x 8 | |
| 15 | TRINITY_DN6 | −0.04 | 4e−12 | 3e−11 | 5 x 6 | |
| 16 | TRINITY_DN1 | −0.03 | 4e−12 | 4e−11 | 5 x 13 | |
| 17 | TRINITY_DN1 | −0.07 | 5e−12 | 4e−11 | 3 x 4 | |
| 18 | TRINITY_DN1 | −0.07 | 5e−12 | 4e−11 | 3 x 4 | |
| 19 | TRINITY_DN6 | −0.09 | 6e−12 | 1e−10 | 13 x 14 | |
| 20 | TRINITY_DN6 | −0.08 | 7e−12 | 1e−10 | 11 x 17 | |

**Portrait**

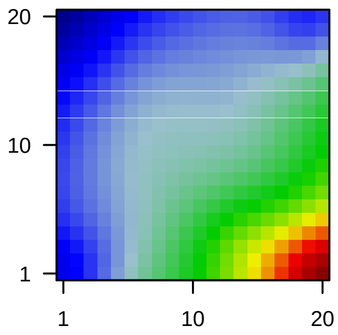


**Top 100 DE genes**

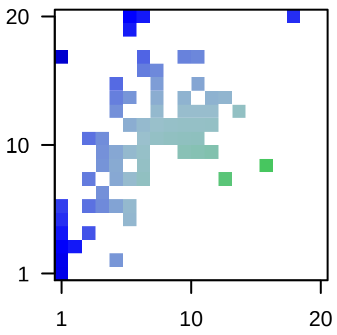


**p−values**

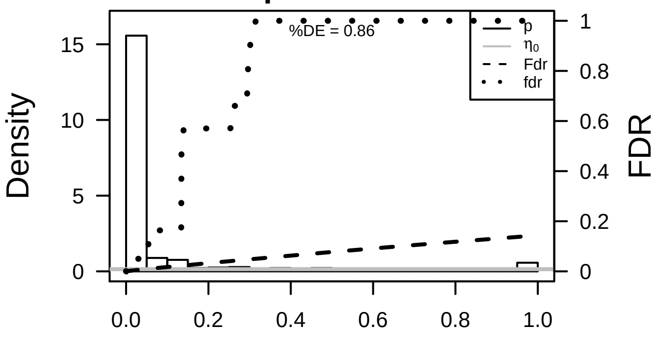

Supplement: Supplementary file 1 [file cells-09-00779-s001.zip › Supplementary materials/FileS3.zip!/FileS2/Hormones - Results/Summary Sheets - Groups/Reports/Zhongwan.6.10..pdf]
